# Anion-Specific Adsorption of Carboxymethyl Cellulose on Cellulose

**DOI:** 10.1021/acs.langmuir.3c01924

**Published:** 2023-10-11

**Authors:** Vishnu Arumughan, Hüsamettin Deniz Özeren, Mikael Hedenqvist, Marie Skepö, Tiina Nypelö, Merima Hasani, Anette Larsson

**Affiliations:** † Department of Chemistry and Chemical Engineering, Chalmers University of Technology, SE-41296 Gothenburg, Sweden; ‡ AvanCell, Chalmers University of Technology, SE-41296 Gothenburg, Sweden; § School of Engineering Sciences in Chemistry, Biotechnology and Health, Polymeric Materials Division, Fiber and Polymer Technology, KTH Royal Institute of Technology, SE-100 44 Stockholm, Sweden; ∥ Wallenberg Wood Science Center, KTH Royal Institute of Technology, SE-100 44 Stockholm, Sweden; ⊥ FibRe Vinnova Competence Center, KTH Royal Institute of Technology, SE-100 44 Stockholm, Sweden; # Division of Theoretical Chemistry, Lund University, P. O. Box 124, SE-221 00 Lund, Sweden; ∇ Wallenberg Wood Science Center, Chalmers University of Technology, SE-41296 Gothenburg, Sweden; ○ FibRe Vinnova Competence Center, Chalmers University of Technology, SE-41296 Gothenburg, Sweden

## Abstract

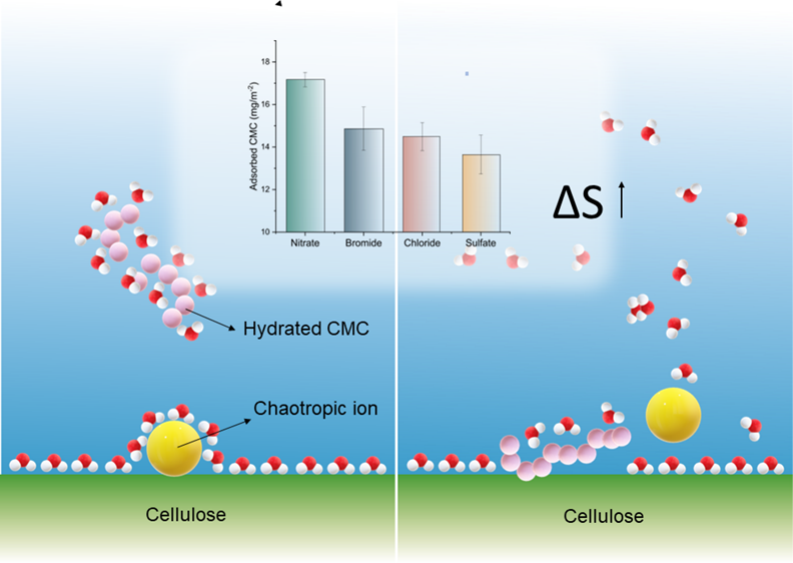

Integration of fiber modification step with a modern pulp mill is a resource efficient way to produce functional fibers. Motivated by the need to integrate polymer adsorption with the current pulping system, anion-specific effects in carboxymethylcellulose (CMC) adsorption have been studied. The QCM-D adsorption experiments revealed that CMC adsorption to the cellulose model surface is prone to anion-specific effects. A correlation was observed between the adsorbed CMC and the degree of hydration of the co-ions present in the magnesium salts. The presence of a chaotropic co-ion such as nitrate increased the adsorption of CMC on cellulose compared to the presence of the kosmotropic sulfate co-ion. However, anion-specificity was not significant in the case of salts containing zinc cations. The hydration of anions determines the distribution of the ions at the interface. Chaotropic ions, such as nitrates, are likely to be distributed near the chaotropic cellulose surface, causing changes in the ordering of water molecules and resulting in greater entropy gain once released from the surface, thus increasing CMC adsorption.

## Introduction

Surface modification of cellulose fibers via polyelectrolyte adsorption is of substantial interest as it paves the way to a multitude of highly relevant applications ranging from specialty papers to paper-based diagnostics.^1−5^ Cationic polyelectrolytes can readily be adsorbed on cellulose as the surface is negatively charged due to the residual amount of charged hemicellulose.^6−8^ Notably, negatively charged polyelectrolyte systems such as PEDOT: PSS and carboxymethyl cellulose (CMC) were also found to have affinity toward cellulose surface.^1,9−11^ The adsorption of CMC on cellulose has been used as a resource-efficient way to introduce surface charges. The presence of carboxylic groups on the surface enhances the swelling of the fiber, thus improving cell wall cohesion and increasing strength properties in final paper sheets.^2^

The seminal work by Laine et al.^2,11^ elucidated the controlling factors of CMC adsorption. The addition of salts can modulate the adsorption of negatively charged polyelectrolytes. According to Fleer et al.,^12^ the charges on negatively charged surfaces are screened with the addition of salt, and nonelectrostatic factors drive adsorption of anionic polyelectrolytes. Divalent cations are more effective in increasing adsorption as they can efficiently screen surface charge and induce favorable structural changes in the polyelectrolyte solution for adsorption.^13,14^ However, the influence of ions on different processes is governed not just by their charge. Factors such as ionic radii and ion hydration become more decisive, especially in interfacial processes such as adsorption. Recently, it was reported that adsorption of CMC is prone to cation-specific effect, where adsorption of CMC in the presence of Ca^2+^ was higher than in the presence of Mg^2+^ ions, and the observed change in adsorbed CMC was ascribed to dispersion interactions due to the larger polarizability of Ca^2+^ ions.^10^

Changes in observed properties in biological and nonbiological systems stemming from ions’ identity are broadly called ion-specific effects.^15^ The majority of the research on specific ionic effects on interfacial phenomena exclusively investigates the effects of counterions, while the effect of different co-ions is often overlooked, because co-ions are anticipated to be excluded from the interface according to the Debye–Huckel theory. There are only a few studies that have looked into the effects of co-ions.^16,17^ One of the first report on co-ion specificity was by Ninham and co-workers^18^ where they demonstrated anion-specific effects on negatively charged glass electrodes. The pH values were found to be highly dependent on the co-ions present in the background electrolytes.^18,19^ Recently, Sthoer et al.^16^ have shown that the identity of the anions has a significant effect on the charging behavior and degree of deprotonation of the carboxylic acid group-containing Langmuir monolayers. Co-ions may play a substantial role in interfacial interactions involving negatively charged surfaces, such as cellulose. Mittal et al.^17^ demonstrated that the specific ion characteristics such as hydration enthalpy and polarizability of co-ions govern the mechanical properties of highly oriented cellulose microfibers prepared via flow focusing method.

To the best of our knowledge, anion-specific effects in polyelectrolyte adsorption on cellulose surfaces have not been studied so far. Besides their scientific significance, anion specificities in anionic polyelectrolyte adsorption on cellulose could have significant industrial implications. Magnesium ions have been recommended as an alternate adsorption enhancer to calcium ions in efforts to integrate CMC adsorption with present pulp mills.^10^ This is because the presence of Ca^2+^ ions in the pulping mill results in scaling issues caused by the deposition of calcium oxides and calcium carbonates, which are undesirable in the industry. Moreover, magnesium sulfate is deliberately being added to the oxygen delignification stage to preserve carbohydrate yield. However, when we compare the ability of Ca^2+^ and Mg^2+^ ions to improve the adsorption of CMC under the same conditions, Mg^2+^ ions are not as good as Ca^2+^.^10^ We anticipate that, if CMC adsorption is prone to anion-specific effects, it could be possible to improve adsorption of CMC by choosing the right combination of Mg^2+^ and co-ion.

In this investigation, the aim was to reveal the effect of anions on the adsorption of CMC on cellulose surface. Adsorption of CMCs in the presence of different magnesium salts was studied by using a Quartz Crystal Microbalance with Dissipation (QCM-D). The adsorption studies revealed that the adsorption of CMC is prone to co-ion specific effects, the hydration of co-ions has found to be an important factor that determines the interaction of CMC with the cellulose surface. We anticipate that the findings from this investigation contribute to a fundamental knowledge of the ion specificities involved in polymer adsorption on cellulose surfaces and the development of resource-efficient industrial processes to produce new fibers.

## Experimental Section

### Materials

The cellulose nanofibers (CNF) with an average diameter of 5 nm and carboxylic content of 31.4 μmol/g from softwood Kraft fibers were obtained from Stora Enso, Sweden. The nanocellulose fibers used in this study were produced via enzymatic treatment combined with mechanical disintegration. It have a residual hemicellulose content of 14.7% (xylose 8%, arabinose 0.62%, galactose 0.25% and mannose 6.1%) and lignin content of 1.1% (Klasson lignin 0.35% and acid-soluble lignin). Magnesium chloride (MgCl_2_), magnesium bromide (MgBr_2_), magnesium nitrate (Mg(NO_3_)_2_), magnesium sulfate (MgSO_4_), zinc nitrate (Zn(NO_3_)_2_), and zinc sulfate (ZnSO_4_) were purchased from Sigma-Aldrich. Carboxymethyl cellulose (CMC), more precisely Blanose 7LPEP with a molecular weight of 90.5 kDa and a degree of substitution (DS) of 0.7 (according to the supplier), was kindly provided by Ashland, Sweden

### Methods

#### Preparation and Characterization of Ultrathin Cellulose Model Film for the Adsorption Studies

A cellulose model film was prepared according to the protocol described in our previous report.^10^ A CNF suspension of concentration 1.7 g/L was sonicated and centrifuged, and the supernatant was collected for spin coating. SiO_2_ QCM-D sensors supplied by Q-Sense AB (Gothenburg, Sweden) were coated with an anchoring layer of polyethylenimine (PEI) by immersing the sensors in a PEI solution (1.6 g/L) for 20 min. The prepared CNF suspension were then spin coated on PEI coated QCM sensors using following spinning parameters: 3000 rpm, acceleration of 2100 rpm/s for 1 min. The spin coated QCMD surfaces were then dried in an oven at 80 °C for 10 min and stored in the desiccator with silica gel.

The film morphology and uniformity were analyzed using atomic force microscopy (INTEGRA Prima setup NT-MDT Spectrum Instruments, Moscow, Russia). In addition, height profiles of 3 random spots on the film were recorded in semicontact mode, and the root means square roughness was calculated using Gwydion software to assess the quality of the film.

The water content of the CNF model film was calculated according to the procedure reported by Kittle et al.^20^ using a QCM-D instrument (Biolin Scientific, Gothenburg, Sweden). In this procedure, the QCM-D sensors coated with CNFs were placed in the flow cell, and deionized water was injected into the flow cell at the rate of 100 μL per minute for 3 h to get a stable baseline. Then the solvent was switched to D_2_O, and the response was recorded until a plateau in the frequency shift occurred. After that, the solvent was switched back to H_2_O again. Calculation of the water contained in the film was based on the advantage of the density difference between H_2_O and D_2_O.

#### Zeta Potential Determination

The effect of anions on the zeta potential CNF (in suspension) was analyzed using Zeta sizer Nano Zs (Malvern Instruments, UK). A 0.5% (w/v) CNF suspension was probe sonicated in an ice bath for 1 min and diluted ten times to get a CNF suspension of concentration 0.05% (w/v); the electrolyte concentration of the suspension was adjusted by adding an appropriate amount of 1 M corresponding salt solutions.

#### Adsorption Experiments using QCM-D

The co-ion specificities in CMC adsorption were evaluated by performing model adsorption experiments using QCM-D (QCM-D E4, Biolin scientific, Gothenburg, Sweden). All of the experiments were performed at 25 °C with a flow rate of 100 μL/min. CMC solutions (concentration of 0.2% (w/v)) containing different salts (MgCl_2_, MgBr_2_, Mg (NO_3_)_2_, MgSO_4,_ Zn(NO_3_)_2,_ and ZnSO_4_) were injected into the flow cells. The CNF film was equilibrated with the corresponding buffer solutions prior to the injection of the CMC solution.

The adsorbed mass was calculated using a model developed by Johannsmann et al.^21^ This equation relates the shift in the complex frequency to the crystal’s resonance frequency in solution:
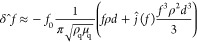
1where δ̂*f* is the shift in the complex frequency, *f*_0_ is the fundamental resonance frequency of the quartz crystal in air, *f* is the resonance frequency of the crystal in contact with the solution, *d* is the thickness of the film, and *ĵ*(*f*) is the complex shear compliance. ρ_q_ and μ_q_ are the specific density and elastic shear modulus of the quartz crystal, respectively. Equation 1 can be written in a simpler form by using equivalent mass (m^*^), which is defined as
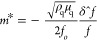
2and then, we obtain a linear equation


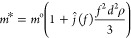
3It is assumed that *ĵ*(*f*) is independent of frequency in the accessible range, and the true sensed mass *m*^o^ is obtained graphically by plotting the equivalent mass against the square of the resonance frequency. In this investigation, third, fifth, and seventh overtones were used for Johannsmann’s modeling. It is important to mention that the true sensed mass calculated using Johannsmann’s modeling includes the mass of water that is associated with the adsorbed layer and thus not equal to the dry mass of adsorbed CMC.

## Results and Discussion

**Figure 1:**
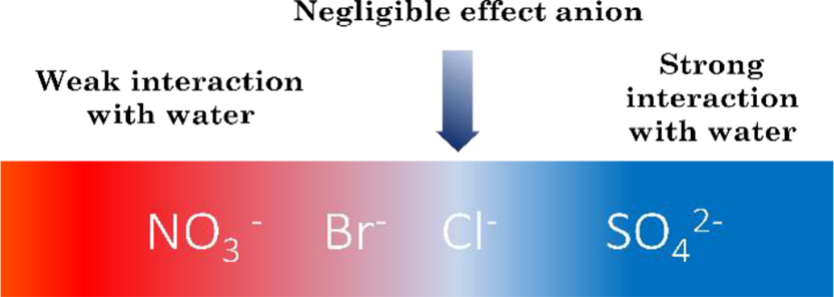
Hydration characteristics of anions.

To begin with, Figure 1 illustrates the hydration properties of four anions used in this investigation. Cl^–^ is frequently seen as an ion with negligible effect in the context of the Hofmeister effect, and it is usually considered as a reference point in the Hofmeister series.^22^ The anions on the left of Cl^–^ are less hydrated and are commonly referred to as chaotropes. On the other hand, the anion to the right of Cl^–^, in this case, SO_4_^2–^, is highly hydrated and referred to as kosmotrope.

**Figure 2:**
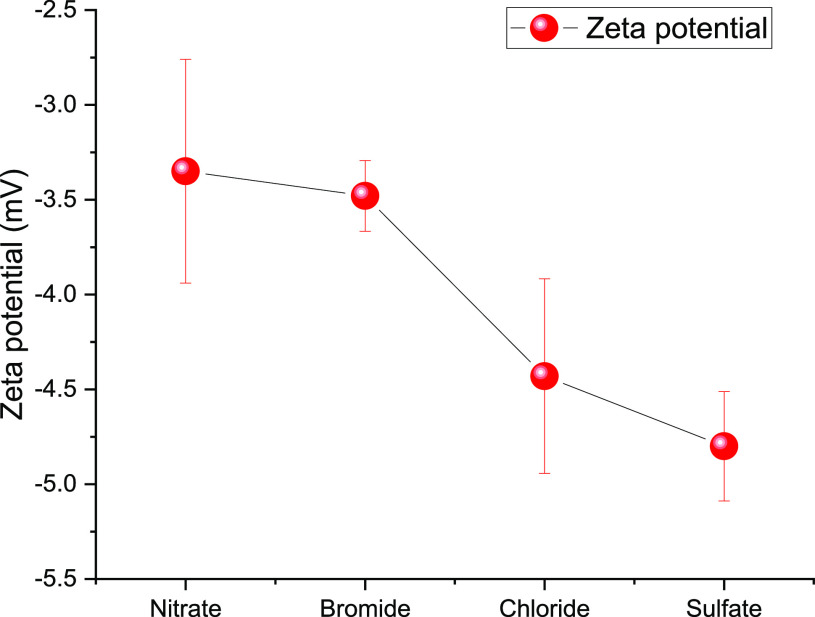
Zeta potential of CNF suspension in magnesium salts containing different co-ions.

Anion-specific effects on the surface of CNFs were evaluated using zeta potential measurements in the presence of 0.02 M magnesium salts containing chloride, bromide, nitrate, and sulfate. When no salt was added, the CNF suspension had a zeta potential of −35 mV, indicating that the suspension is colloidally stable. The surface charge density of the CNF used in this study was 31 μmol/g. Since the CNF samples were not oxidized using TEMPO oxidation or carboxymethylation, these surface charges are likely to be generated due to the presence of hemicelluloses. Figure 2 shows the zeta potential values of CNF suspensions in magnesium salt solutions with different co-ions.

The addition of salt shifted the zeta potential to a less negative value, meaning that the charges were screened. Interestingly, the zeta potential values of CNF showed slight changes depending on the co-ion, where the presence of magnesium sulfate or magnesium nitrate gave the largest difference. A recent investigation by Simonsson et al.^23^ also reported a similar trend in zeta potentials of silica particles in the presence of sodium salt containing sulfate and nitrate.^23^ The silica particles had a more negative zeta potential in the presence of sulfate co-ion compared to nitrate as the co-ion. Our results presented in Figure 2 indicate that the co-ion identity influences the surface charge of CNF. However, the colloidal stability of the CNF suspension was tampered at 0.02 M concentration of salts, and a quantitatively accurate judgment about co-ion specificity cannot be made based on the zeta potential results.

**Figure 3:**
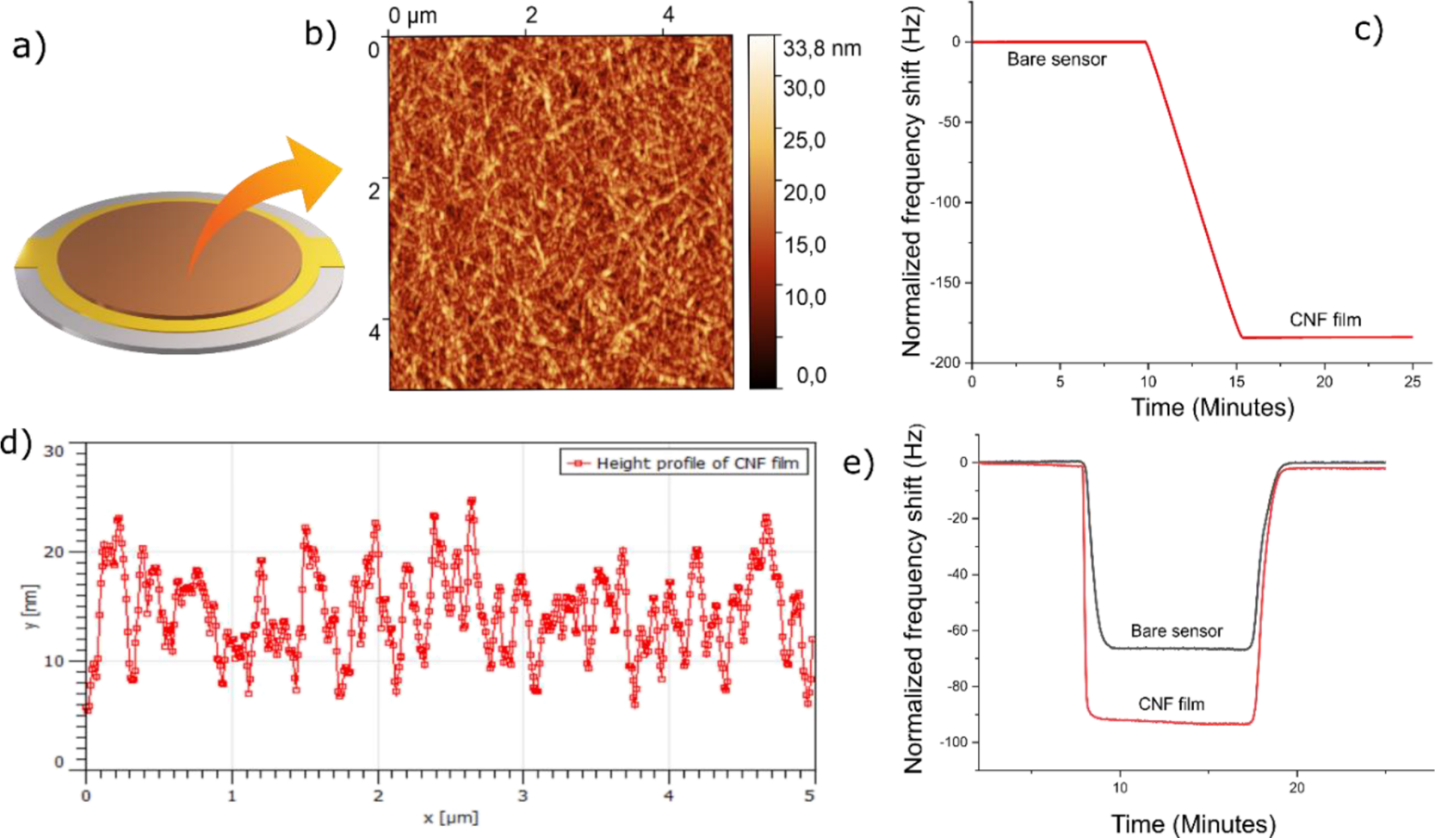
(a) Illustration of CNF coated QCM-D sensor. (b) AFM micrograph of the spin-coated CNF film. (c) Stitched QCM-D data of the bare sensor in air and the CNF coated sensor. (d) Height profile of CNF model film along a horizontal line. (e) Representative QCM-D raw data (3rd overtone) of solvent exchange for a bare sensor (black) and a sensor coated with CNF (red).

To further investigate the effect of co-ions in the adsorption of CMC on cellulose, adsorption experiments were conducted in the presence of a magnesium salt containing different co-ions. Due to the heterogeneity of cellulose fibers in terms of structure and chemistry, fundamental adsorption studies are challenging. To overcome the aforementioned challenge, this study utilized cellulose model surfaces, where CNF suspensions were spin-coated to form ultrathin cellulose films as model cellulose surfaces. Figure 3b shows the AFM micrograph of the CNF model film on a QCM-D sensor. The resonance frequencies of the bare sensor and CNF deposited sensor were measured in the air and presented in Figure 3c. A negative frequency shift was observed corresponding to the areal mass of CNF of 32.2 mg/m^2^. The average thickness of the film was determined to be 21.7 ± 0.8 nm by assuming that the fibrils are perfectly oriented and packed.^24^ However, one should keep in mind that, in reality, CNF forms a diffused network on the sensor surface.^24^

The D_2_O–H_2_O solvent exchange studies revealed that model films contain 40 mg/m^2^ water, indicating that the films are hydrated. The prepared cellulose model surfaces showed a uniform fibrillar morphology with a mean roughness of 3.7 nm; the substrate can be considered ‘smooth’. However, on a molecular level, these surfaces cannot be considered as homogeneous because the CNF has a fibrillar morphology and contains a significant amount of hemicelluloses.

**Figure 4:**
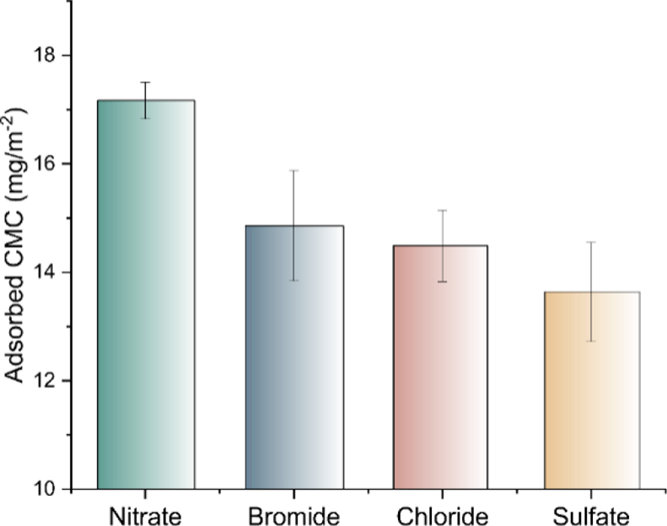
Adsorbed mass of CMC from 0.02 M solutions of magnesium salts with different co-ions.

The adsorption studies have been conducted in the presence of magnesium salts with different co-ions (Mg(NO_3_)_2_, MgBr_2_, MgCl_2,_ and MgSO_4_) at a concentration of 0.02 M (Figure 4). The model surfaces were equilibrated in the corresponding buffer solution and exposed to CMC solutions containing magnesium salts with different co-ions.

The adsorbed mass of CMC was computed using Johannsmann’s model.^21^ The identity of co-ions was found to influence the adsorption of CMC on cellulose. CMC adsorption in the presence of magnesium nitrate was higher than those in other salts studied. The difference in the adsorbed CMC in the presence of magnesium nitrate and magnesium sulfate was more significant. To investigate this further, CMC adsorption was carried out at different magnesium nitrate and magnesium sulfate concentrations (0.002 M – 0.020 M).

**Figure 5:**
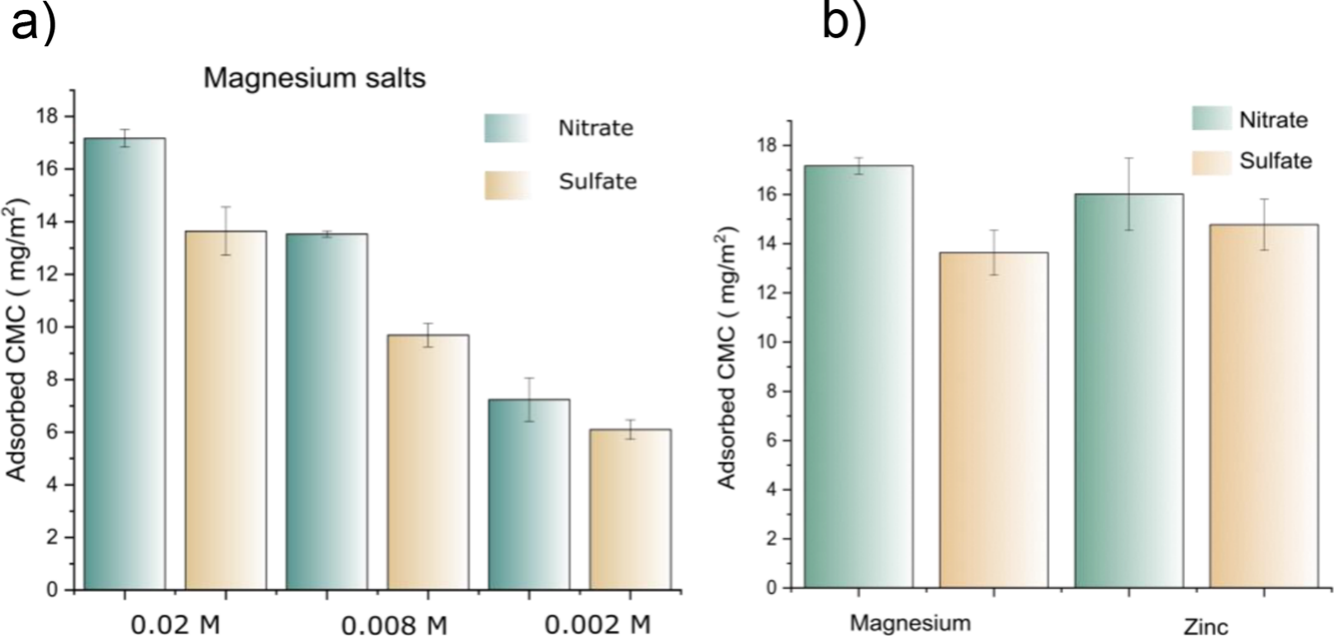
(a) Adsorbed CMC from different concentrations of magnesium nitrate and magnesium sulfate. (b) CMC adsorbed from nitrate and sulfate salts of magenesium and zinc at a concentration of 0.02 M.

Figure 5a shows adsorbed amount of CMC in the presence of different concentrations of MgSO_4_ and Mg(NO_3_)_2_. ANOVA analysis on the data sets have revealed that the differences in adsorption of CMC in the presence of sulfate and nitrate co-anions are significant at 0.02 and 0.008 M. At lowest concentration studied (0.002 M), the difference was not significant. From these observations, it is evident that the CMC adsorption is prone to anion-specific effect; however, at lower concentrations, it is not apparent.

To investigate the effect cations have on the anion-specific character of CMC adsorption, similar adsorption studies were conducted in the presence of zinc sulfate (ZnSO_4_) and zinc nitrate (Zn(NO_3_)_2_). Figure 5b shows the adsorbed CMCs in the presence of sulfate and nitrate salts of magnesium and zinc cations. Zinc salts also show the same trend as seen in the case of magnesium salts; however, the difference was not significant, meaning that the occurrence of co-ion specificity depends on the cation present in the system.

**Figure 6:**
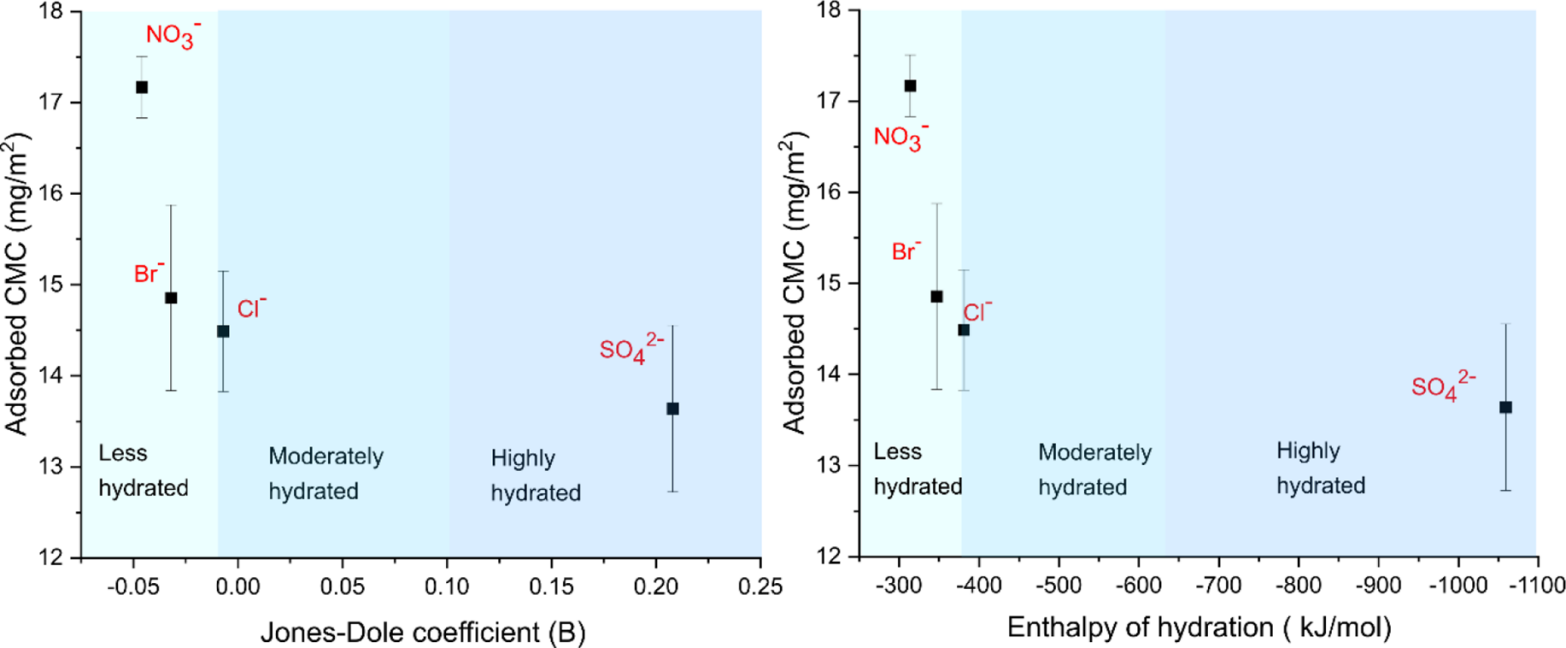
(a) Adsorbed mass of CMC in the presence of 0.02 M magnesium salts plotted against Jones–Dole coefficient. (b) Adsorbed mass of CMC in the presence of 0.02 M magnesium salts plotted against enthalpy of hydration. The values for Jones–Dole coefficient and hydration enthalpy of ions were taken from Robinson et al. and Smith, respectively.^26,27^

Generally, ion-specific effects have been explained in terms of the hydration of the ions. The Jones–Dole viscosity coefficient (B) and hydration enthalpy are two parameters that show the degree of hydration of ions. The positive value of the Jones–Dole coefficient implies that the ion is hydrated; conversely, the negative B values indicate that the ions are less hydrated.^25^ The CMC adsorption data obtained from QCM-D studies were correlated with the Jones–Dole coefficient and enthalpy of hydration of co-ions and presented in Figure 6.

Figure 5 shows that the adsorbed CMC and the degree of hydration of co-ions have a clear correlation. Adsorption is promoted in the presence of a less hydrated (chaotropic) nitrate ion, while adsorption is reduced in the presence of highly hydrated sulfate ions. To explain the ion specificity in adsorption of CMC on the cellulose surface, the CNF surface properties and hydration characteristics should be established. According to Mittal et al.,^17^ the CNF surface should be considered as chaotropic since kosmotropic polymers are soluble in water. The Flory and Huggins interaction parameter is a dimensionless variable that describes the difference in interaction energy between a solvent molecule submerged in a pure polymer and a solvent molecule immersed in a pure solvent. A positive χ parameter implies less polymer–solvent interaction, whereas a negative χ parameter indicates that polymer–solvent interaction is high.^28^ The majority of carbohydrate polymers have a positive χ parameter in water, which means that their interactions with water are often weaker than those between water molecules.^29^ Additionally, while the surface of cellulose is typically considered hydrophilic, the amphiphilic nature of cellulose has been discussed and determined to be responsible for cellulose’s insolubility in water.^30^ The presence of surface charge on cellulose increases the interaction with the solvent; however, the surface charge density of the CNF employed in this study is modest, and these charges will be screened in the presence of salts, further lowering the interaction with water.

According to classical mean-field theory, descriptions of charged surface–liquid interface anions are expected to be repelled from the negatively charged interfaces. At aqueous interfaces, however, ion-specific effects can dominate over direct electrostatic interactions.^31^ Studies on air/liquid interface showed that anions adsorb at negatively charged air/water interface.^32−35^ Large polarizable ions with a smaller hydration shell prefer the hydrophobic air–water interface, whereas small and intermediately polarizable ions with a larger hydration shell prefer to remain in bulk water. According to the literature, the plausible mechanism by which anions adsorb to negatively charged surfaces are the following: (i) The ions with large size and high polarizability can interact with the surface through dispersion.^36,37^ (ii) An adapted version of the classic theory of hydrophobic solvation can be used to explain the ion’s interfacial affinity.^38^ According to this theory, the free energy required to solvate a small neutral cavity is proportional to the cavity volume.^39^ This indicates that the ion’s size mostly determines the adsorption of ions at the air/water interface and hence its hydrophobic character. Additionally, (iii) water’s collective dipole-moment fluctuations are suppressed when it comes into contact with an ion, which exerts a force on the ion, attracting it to regions with a lower density of dipole-moment fluctuations than in bulk water, i.e., regions with a lower relative permittivity.^36,40^

**Figure 7:**
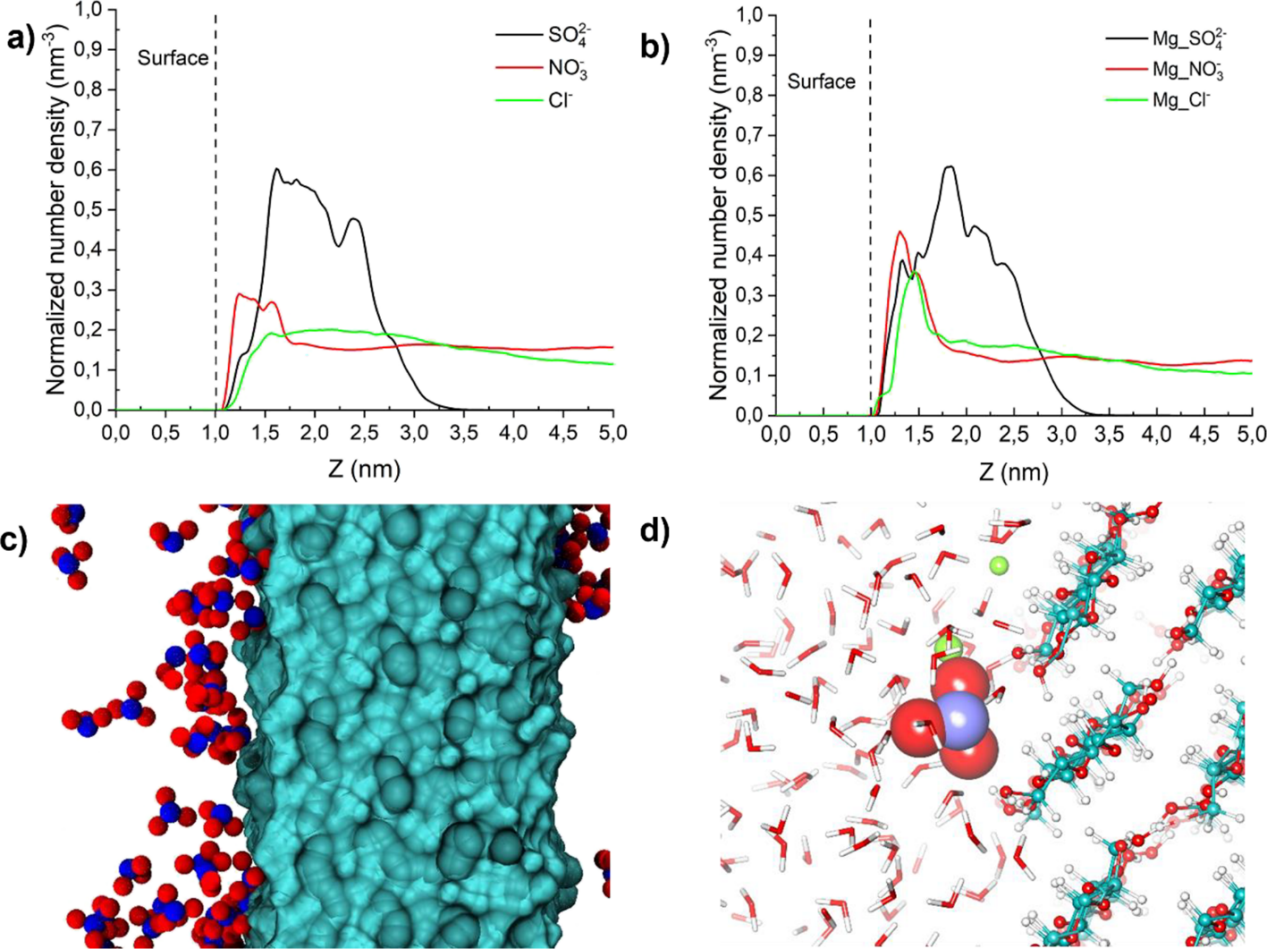
(a) Density profile of anions from the cellulose surface. (b) Density profile of magnesium ions from the cellulose surface in the presence of different coanions. (c) The snapshot from the simulation showing NO_3_^–^ ions around the cellulose surface. (d) A single NO_3_^–^ ion and Mg (green) interaction close to the surface (left). Red, blue, and cyan represent oxygen, nitrogen atoms, and cellulose surface or carbon atom, respectively.

Similarly, in the case of the cellulose-liquid interface, it has been suggested that the chaotropic ions prefer to stay close to a chaotropic surface to reduce the perturbation of the water hydrogen bonding network. Conversely, the kosmotropic ions avoid the vicinity of the chaotropic surface and prefer to stay in the bulk.^17^ So, the less hydrated nitrate ions could preferably accumulate close to the chaotropic CNF surface. Molecular dynamic simulations have been performed to study the distribution of anions at the cellulose-water interface. The normalized number density of the anions in the perpendicular distance from the cellulose slab is given in Figure 7.

From Figure 7a,b, it can be seen that both cations and anions are situated in the vicinity of the cellulose surface. The nitrate ions accumulated at a distance of 2.3 Å, whereas the chloride ions were situated mostly at a distance if 5.3 Å. This suggests nitrate anions are strongly adsorbed to the surface, more than chloride anion. Interestingly, it was observed that the sulfate ions have a tendency to form aggregates at a distance of 15 Å and the normalized number density in the bulk approaches zero. The corresponding density profile of magnesium ions also found to follow the same trend as sulfate ions, which suggest the formation of ion-pair clusters. Hou et al.^41^ has observed similar type of clustering of sodium ions and sulfate ions.

The ion-pairing between anions and cations may affect how an ion interacts with interfaces.^42−44^ According to Simonsson et al.,^23^ the ion-pairing in solution decreases the effective concentration of cations that can be associated with the colloidal surface, in our case this might cause changes in adsorption behavior. The law of water affinities (LWMA) can be used to gain insights into ion pairing. According to LWMA, the ions form stronger contact ion pairs if they have similar hydration behavior so that the hydration shells between the ions can be shared.^42^ From this perspective, sulfate ions with a B-coefficient of 0.208 have hydration behavior similar to that of magnesium ions (B-coefficient = 0.385), and they tend to form stable contact ion-pairs. Magnesium ions could be pulled out from the interface by sulfate ions, reducing the availability of magnesium ions at the interface which is in agreement with the simulation results presented in Figure 7b. In contrast, in the case of nitrate ions (B-coefficient = −0.046) and magnesium ions (B-coefficient = 0.385), the hydration enthalpy significantly differs, indicating that these ions could be acting as well separated ions allowing ‘independent’ mobility. Xu et al.^45^ have reported that the ion-pairing of interfacial nitrate ions and magnesium ions is negligible, and nitrate anions in the air–aqueous interfacial region have been found to be relatively free from Coulombic effects of magnesium ions. This ‘independent mobility of magnesium ions and nitrate ions contribute to their availability at the interface and, thereby, influences CMC’s adsorption to cellulose surfaces

**Figure 8:**
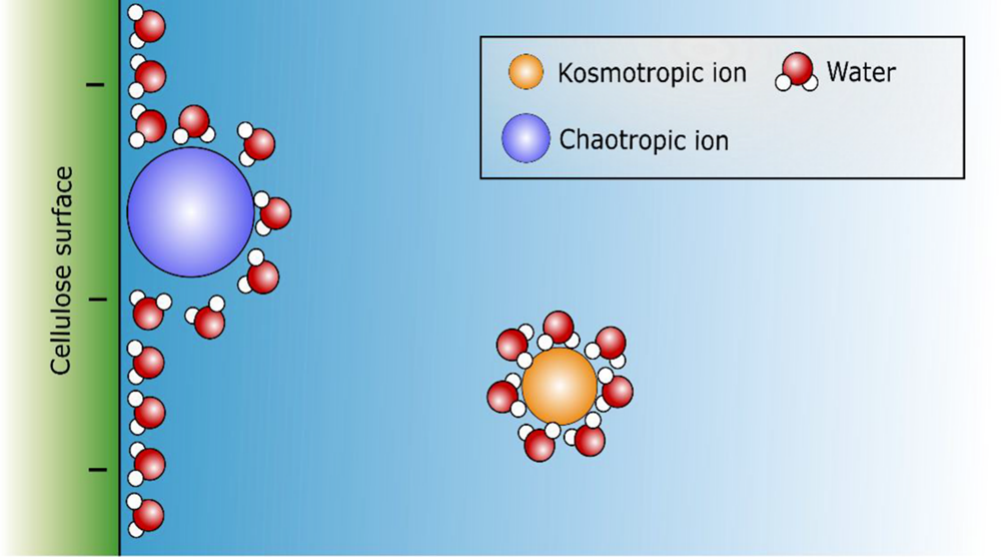
Position of hydration dictated the positioning of nitrate and sulfate ions at the interface. The image is not to the scale.

Figure 8 illustrates the distribution of chaotropic nitrate ions and kosmotropic sulfate ions at the interface.

Since the charges in cellulose nanofibers originated due to hemicellulose, the distribution of these charges will be nonuniform, and we could consider it a patchy surface. The anions might interact with the hydrophobic domains of the cellulose exposed to water, but how could the accumulation of chaotropic anions alter the adsorption of CMC on the cellulose surface? To answer this question, the driving force of the adsorption of polymers should be discussed.

Recently, investigations suggest that the adsorption of polymers on cellulose surfaces is driven by entropy gain due to the release of water that is unfavorably arranged on the surface.^9,13,46−48^ The distribution of ions at a charged interface plays a crucial role in determining the effect of charge on the water organization.^36^ The vicinity of chaotropic nitrate ion at the interface could be causing a more unfavorable organization of water molecules at the interface, which results in larger entropy gain once released from the surface and thus higher adsorption of CMC.

### Conclusions

Previous research on the adsorption of CMC on cellulose has concentrated on the influence of counterions used to enhance adsorption. We investigated the anion-specific effect of CMC adsorption on cellulose. Adsorption studies using QCM-D demonstrated that CMC adsorption is anion specific in the presence of magnesium salts and that adsorption can be modified by alteration of the anions in the salt. There is indeed a correlation between the adsorbed mass of CMC and the degree of hydration of the magnesium salt’s co-ions. In comparison to the presence of a kosmotropic sulfate co-ion, a chaotropic co-ion such as nitrate increased the adsorption of CMC on cellulose. However, in the case of salts containing zinc cations, the anion specificity was not significant.

According to the current understanding of polymer adsorption on cellulose surfaces, adsorption is driven by the entropy gain associated with the release of ordered water molecules from the surface. Therefore, it is critical to address the distribution of anions at the interface and their effect on water structure. While chaotropic ions such as nitrate prefer to remain near the surface of chaotropic cellulose, kosmotropic ions such as sulfate ions prefer to be in bulk. Accumulation of chaotropic ions at the interface may alter the orientation of water molecules, resulting in a greater entropy gain when they are released and enhancing adsorption. CMC adsorption could be incorporated into the current pulping system through the judicious use of co-ion specificity in the pulping process. The co-ion specificity may also have implications for other interfacial phenomena involved in the pulping process, such as lignin diffusion, lignin readsorption, and hemicellulose adsorption. The authors urge further research into these aspects to develop resource-efficient pulping processes.

## Supporting Information Available

Details of molecular dynamic simulation (PDF)
