# Supplementary material for: Anion-Specific Adsorption of Carboxymethyl Cellulose on Cellulose
Source: Langmuir. 2023 Oct 11;39(42):15014–21. doi: 10.1021/acs.langmuir.3c01924 (PMC10601536; doi:10.1021/acs.langmuir.3c01924)
Supplement: Supplementary file 1 — la3c01924_si_001.pdf [file la3c01924_si_001.pdf]

Anion specific adsorption of carboxymethyl cellulose on cellulose

Vishnu Arumughan[1,2], Hüsamettin Deniz Özeren[3], Mikael Hedenqvist[3,4,5], Marie Skepö[6], Tiina Nypelö[1,7], Merima Hasani[1,2,7] & Anette Larsson[1,2,7,8]**

1. Department of Chemistry and Chemical Engineering, Chalmers University of Technology, SE-41296, Gothenburg, Sweden
2. AvanCell, Chalmers University of Technology, SE-41296, Gothenburg, Sweden
3. KTH Royal Institute of Technology, School of Engineering Sciences in Chemistry, Biotechnology and Health, Polymeric Materials Division, Fiber and Polymer Technology, SE-100 44, Stockholm, Sweden
4. Wallenberg Wood Science Center, KTH Royal Institute of Technology, SE-100 44, Stockholm, Sweden
5. FibRe Vinnova competence center, KTH Royal Institute of Technology, SE-100 44, Stockholm, Sweden
6. Division of Theoretical Chemistry, Lund University, P.O. Box 124, SE-221 00 Lund, Sweden
7. Wallenberg Wood Science Center, Chalmers University of Technology, SE-41296, Gothenburg, Sweden
8. FibRe Vinnova competence center, Chalmers University of Technology, SE-41296, Gothenburg, Sweden

Corresponding authors: Dr. Vishnu Arumughan (vishnu.arumughan@chalmers.se), Prof. Anette Larsson (anette.larsson@chalmers.se)

1. Molecular dynamics simulations

1.1. Cellulose surface and solvent

The surfaces were built as one slab, four layers thick, where each chain was 16 anhydroglucose units long, exposing the Iβ 1-10 crystallographic plane using Cellulose-builder [1]. A total of 25 units of the upper and lower surface was randomly carboxylated to resemble the experimental charge density using Discovery Studio Visualizer 4.5 [2]. The surface was aligned to the z-axis and restrained in a cell with 90 x 86 x 114 Å, and the CHARMM36 force field was chosen for the surface parameters. Subsequently, the cell was solvated with ~ 21000 water molecules, parameterized with the TIP3P force field. The solvated systems were constructed with the periodic boundary that provides infinite size for the surface edge interactions. The anions (Cl^-, NO_3^-, SO_4^{2-}) with the concentration of 0.02 M and Mg^{2+} were dispersed well into the solvated

systems in order to investigate their effects. While Cl^- was parametrized directly from the CHARMM36 force field, those of SO_4^{2-} and NO_3^- were implemented from literature. [3-5]

1.2. Simulation details

The GROMACS 5.1.4 molecular dynamics simulation package [6] was used to carry out all MD simulations. The cutoff distances for all interactions were set to 12 Å for pairwise and electrostatic energy calculations. The hydrogen bonds in the systems were constrained using the LINCS function[7] with the order of four. All the simulations were run with 1 fs timestep and at 1 atm, and long-range electrostatic contributions were calculated with Particle-Mesh Ewald summation with an accuracy of 10^{-4}. [8, 9] The equilibration of the systems after minimization was performed under the NVT (10 ns) at 298 K, NPT (5 ns) at 600 K, and NPT (5 ns) at 300 K with the Berendsen thermostat [10] for pressure and temperature. The initial velocities of all atoms in the equilibration stage were randomly selected from a uniform distribution. The densities and all energies were continuously monitored to ensure that the system was equilibrated enough before starting the production run. The production run consisted of a 100 ns NPT run at 298 K with a Parrinello-Rahman barostat [11, 12] and velocity-rescale thermostat [13] for pressure and temperature, respectively.

The density profile along the z-axis of the cell was generated from the average of the last 20 ns of the production run. The calculation of density was achieved using the distance of the mass center of anions, cations, and the surface of the cellulose. Also, the cellulose-water-anion interactions were investigated using the radial distribution function (RDF). RDF can be defined as the probability of having a B atom located at a distance r from the A atom:

$$g_{A-B}(r) = \frac{V \quad n_B}{N_B 4\pi r^2 dr}$$

where N_B is the total number of B atoms, n_B is the total number of B atoms at a distance r from the A atom (in a shell), and V is the system's total volume. Subsequently, the RDFs were calculated using the cellulose’s oxygen atoms, waters’ hydrogen atoms, and ions.

References

1. Gomes, T. C. F.; Skaf, M. S., Cellulose-Builder: A toolkit for building crystalline structures of cellulose. *Journal of Computational Chemistry* **2012,** *33* (14), 1338-1346.
2. BIOVIA; Systèmes, D. *Discovery Studio Visualizer 4.5*, Dassault Systèmes: San Diego, 2015.
3. Papoyan, G. A.; Gu, K.-j.; Wiórkiewicz-Kuczera, J.; Kuczera, K.; Bowman-James, K., Molecular Dynamics Simulations of Nitrate Complexes with Polyammonium Macrocycles: Insight on Phosphoryl Transfer Catalysis. *Journal of the American Chemical Society* **1996,** *118*, 1354-1364.

4. Cannon, W. R.; Pettitt, B. M.; McCammon, J. A., Sulfate Anion in Water: Model Structural, Thermodynamic, and Dynamic Properties. *The Journal of Physical Chemistry* **1994,** *98* (24), 6225-6230.

5. Vchirawongkwin, V.; Rode, B. M.; Persson, I., Structure and Dynamics of Sulfate Ion in Aqueous SolutionAn <i>ab initio</i> QMCF MD Simulation and Large Angle X-ray Scattering Study. *The Journal of Physical Chemistry B* **2007,** *111* (16), 4150-4155.

6. Abraham, M. J.; Murtola, T.; Schulz, R.; Páll, S.; Smith, J. C.; Hess, B.; Lindahl, E., GROMACS: High performance molecular simulations through multi-level parallelism from laptops to supercomputers. *SoftwareX* **2015,** *1-2*, 19-25.

7. Hess, B.; Bekker, H.; Berendsen, H. J. C.; Fraaije, J. G. E. M., LINCS: A linear constraint solver for molecular simulations. *Journal of Computational Chemistry* **1997,** *18* (12), 1463-1472.

8. Darden, T.; York, D.; Pedersen, L., Particle Mesh Ewald - an N.Log(N) Method for Ewald Sums in Large Systems. *Journal of Chemical Physics* **1993,** *98* (12), 10089-10092.

9. Essmann, U.; Perera, L.; Berkowitz, M. L.; Darden, T.; Lee, H.; Pedersen, L. G., A Smooth Particle Mesh Ewald Method. *Journal of Chemical Physics* **1995,** *103* (19), 8577-8593.

10. Berendsen, H. J. C., Transport Properties Computed by Linear Response through Weak Coupling to a Bath. In *Computer Simulation in Materials Science: Interatomic Potentials, Simulation Techniques and Applications*, Meyer, M.; Pontikis, V., Eds. Springer Netherlands: Dordrecht, 1991; pp 139-155.

11. Parrinello, M.; Rahman, A., Polymorphic Transitions in Single-Crystals - a New Molecular-Dynamics Method. *Journal of Applied Physics* **1981,** *52* (12), 7182-7190.

12. Nosé, S., A molecular dynamics method for simulations in the canonical ensemble. *Molecular Physics* **1984,** *52* (2), 255-268.

13. Bussi, G.; Donadio, D.; Parrinello, M., Canonical sampling through velocity rescaling. *Journal of Chemical Physics* **2007,** *126* (1).
